# Interfering with CSE1L/CAS inhibits tumour growth via C3 in triple‐negative breast cancer

**DOI:** 10.1111/cpr.13226

**Published:** 2022-04-10

**Authors:** Mei Ye, Yufei Chen, Jianni Liu, Jiawei Tian, Xunda Wang, Kin Lam Fok, Jianwu Shi, Hao Chen

**Affiliations:** ^1^ Institute of Reproductive Medicine, Medical School Nantong University Nantong China; ^2^ Xiangyang Central Hospital Affiliated Hospital of Hubei University of Arts and Science Xiangyang China; ^3^ School of Biomedical Sciences, Faculty of Medicine The Chinese University of Hong Kong Hong Kong SAR China

## Abstract

Triple‐negative breast cancer (TNBC) is the most aggressive subtype of breast cancer. However, the treatment regimens for TNBC are limited. Chromosome segregation 1‐like (CSE1L), also called cellular apoptosis susceptibility protein (CAS), is highly expressed in breast cancer and plays a crucial role in the progression of various tumours. However, the involvement of CAS in TNBC remains elusive. In this study, we showed that the expression of CAS was higher in TNBC samples than in non‐TNBC samples in the Gene Expression Omnibus database. Knockdown of CAS inhibited MDA‐MB‐231 cell growth, migration and invasion. Further RNA‐seq analysis revealed that complement pathway activity was significantly elevated. Of note, complement component 3 (C3), the key molecule in the complement pathway, was significantly upregulated, and the expression of C3 was negatively correlated with that of CAS in breast cancer. Lower C3 expression was related to poor prognosis. Interestingly, the expression level of C3 was positively correlated with the infiltration of multiple immune cells. Taken together, our findings suggest that CAS participates in the development of TNBC through C3‐mediated immune cell suppression and might constitute a potential therapeutic target for TNBC.

To the Editor,

Breast cancer is the most commonly diagnosed cancer worldwide. Breast cancer is classified into four molecular subtypes—luminal A, luminal B, Erb‐B2 overexpressing and basal‐like—based on gene expression profiling. The majority of triple‐negative breast cancers (TNBCs),^1^ which are the most aggressive breast cancers and account for 10%–15% of breast cancers, are of the basal‐like subtype. TNBC is characterized by loss of estrogen receptor, progesterone receptor and human epidermal growth factor receptor 2 expression. It has an early onset in young women under 40 years old. Despite the use of chemotherapy as the primary treatment strategy, the 5‐year survival rate of TNBC is poor due to relapse and metastasis.^2^

Chromosome segregation 1‐like (CSE1L), also called cellular apoptosis susceptibility protein (CAS), is involved in nuclear transport. During the shuttling of proteins between the nucleus and the cytoplasm, CAS recycles importin‐α from the nucleus to the cytoplasm for its reuse in a new cycle of protein import.^3^ This activity facilitates the nuclear import a number of cargo proteins that are known to maintain the repression of several methylated genes, thereby silencing the expression of these genes.^4^ CAS also contributes to cell proliferation, apoptosis, migration and cell cycle regulation in multiple cancers.^5^, ^6^ The CAS expression level is also associated with the clinical outcome of cancers.^7^ Although several studies have demonstrated the involvement of CAS in breast cancer development,^7^, ^8^ its role in TNBC remains elusive.

Our previous study found that CAS is highly expressed in breast cancers.^7^ In the present study, to further investigate the role of CAS in TNBC, we examined the mRNA expression of CAS in TNBC using three datasets from the Gene Expression Omnibus database. The expression of CAS was significantly higher in TNBC than in other breast cancer types (Figure 1A, Figure S1A,B).

**FIGURE 1:**
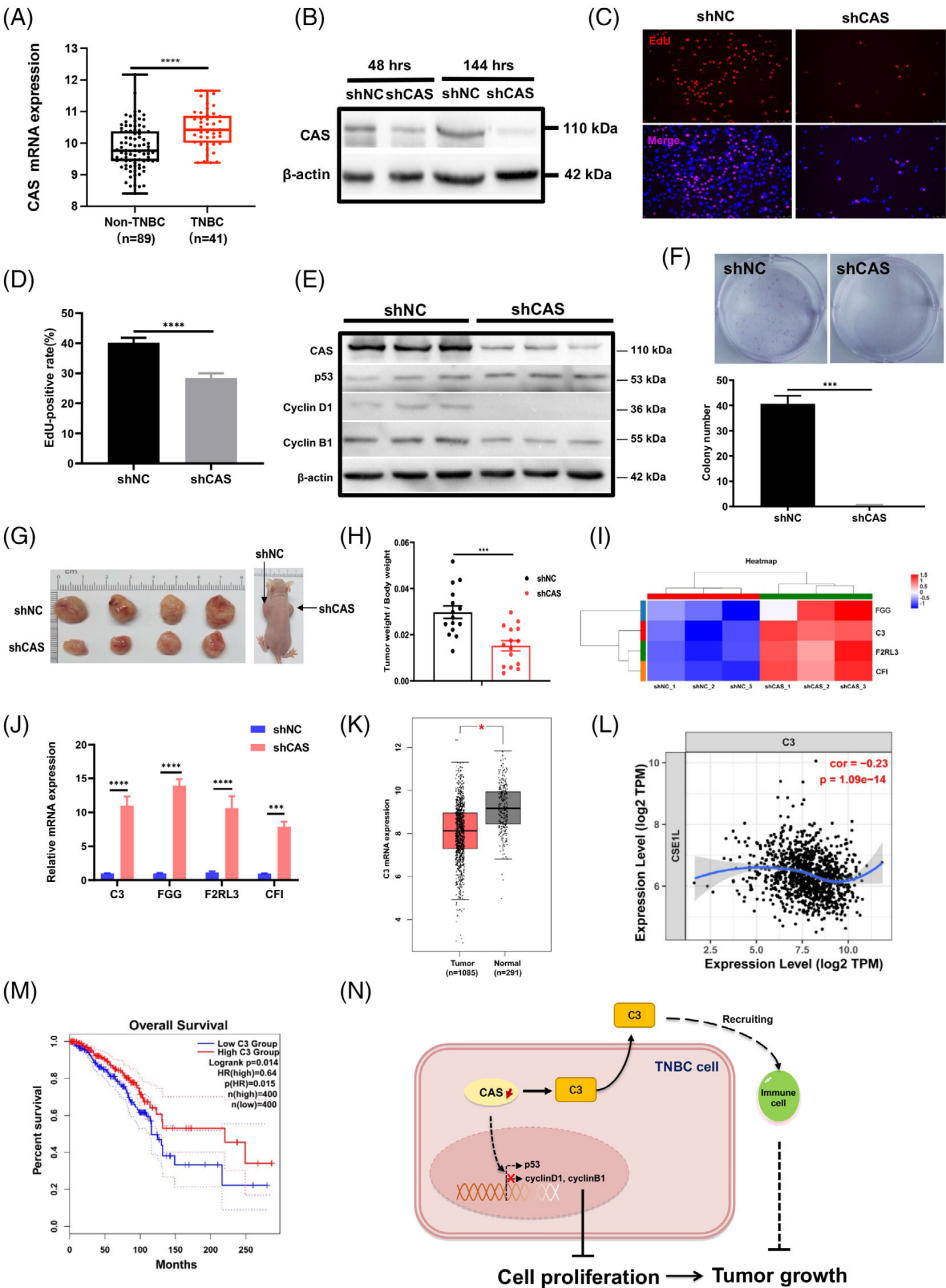
Cellular apoptosis susceptibility (CAS) knockdown inhibited cell growth via the C3 pathway and was associated with the prognosis of triple‐negative breast cancer (TNBC). (A) Comparison of the mRNA expression level of CAS between TNBC and non‐TNBC samples in the GSE65194 dataset (Probe ID: 201111_at). (B) Representative Western blot of CAS knockdown after lentiviral transduction for 48 and 144 h. shCAS, CAS knockdown; shNC, scramble control. (C) Representative images of the EdU incorporation assay 168 h post transfection. (D) Statistical analysis of MDA‐MB‐231 cell proliferation as evaluated by an EdU incorporation assay. The values are presented as the mean ± SEM (****P < 0.0001). (E) Western blot analysis of the expression levels of cell cycle proteins in CAS knockdown cells. (F) Colony formation assay to determine the effects of CAS knockdown in MDA‐MB‐231 cells. The values are presented as the mean ± SEM (***P < 0.001). (G) Representative images of tumours in female BALB/c nude mice bearing MDA‐MB‐231 cells expressing shNC and shCAS (left) and a photograph of the xenograft mouse model (right). (H) Decreased tumour index (tumour weight/body weight) in the shCAS group. The values are presented as the mean ± SEM (***P < 0.001). (I) Heatmap analysis of genes related to complement and coagulation cascades. shCAS, CAS knockdown; shNC, scramble control. (J) Real‐time PCR validation of the RNA‐seq results. (K) Expression analysis of C3 in breast cancer using the GEPIA2 database. (L) Correlation analysis of C3 expression and CAS expression in breast cancer using the TIMER database. (M) Kaplan–Meier analysis of overall survival for breast cancer patients with high (red line, *n* = 400) and low (blue line, *n* = 400) C3 mRNA expression as determined in the GEPIA2 database. (N) Schematic diagram of CAS regulates cell proliferation and C3 singling pathway in TNBC cells

To investigate the functions of CAS in TNBC, we stably knocked down CAS in MDA‐MB‐231 cells by lentiviral transduction of CAS shRNA (shCAS) or scramble control shRNA (shNC) (Figure 1B). The CCK8 assay results showed that knockdown of CAS significantly inhibited the growth of MDA‐MB‐231 cells, with a comparable number of viable cells 168 hours after transduction (Figure S1C). The growth arrest in CAS knockdown cells was attributed to the decrease in cell proliferation, as reflected by the significant decrease in EdU incorporation assay (Figure 1C,D). It is well known that disruption of the cell cycle leads to cell proliferation arrest. We further analysed the expression levels of key cell cycle regulators, such as p53, cyclin D1 and cyclin B1.^9^ We found that in the shCAS group, p53 was significantly upregulated 6 days after transduction (Figure 1E, Figure S1D), while the expression of cyclin D1 and cyclin B1 was significantly decreased, indicating cell cycle arrest. In line with the growth arrest, knockdown of CAS abolished colony formation (Figure 1F). Of note, knockdown of CAS in MCF‐7 cells, derived from another subtype of breast cancer, had no effect on the expression of cyclin B1 in our previous study^7^ or on the expression of p53 in a study by Tanaka et al.^10^ The differential response of cyclins and p53 toward knockdown of CAS suggested the differential roles of CAS in cell proliferation in TNBC compared to non‐TNBCs.

Since CAS is also known to promote metastasis in melanoma,^11^ we examined the migration and invasion of CAS knockdown MDA‐MB‐231 cells. The results showed that loss of CAS significantly inhibited migration and invasion (Figure S1E–G). To further evaluate the effect of CAS on the tumorigenicity of TNBC cells in vivo, shNC or shCAS MDA‐MB‐231 cells were injected into the bilateral armpits of athymic nude mice. The tumour volume was significantly decreased in the shCAS group (Figure 1G,H, Figure S1H). These results indicated that CAS plays an important role in the growth, metastasis and tumorigenicity of TNBC cells.

To decipher the molecular mechanisms underlying the regulation of tumour progression by CAS in TNBC, we performed RNA sequencing to analyse the transcriptome of CAS knockdown MDA‐MB‐231 cells. A total of 8325 differentially expressed genes with a fold change ≥2 and *P* value < 0.05 were identified: 3970 genes were upregulated and 4355 were downregulated in the shCAS group compared to the shNC group (Figure S2A). We subjected differentially expressed genes with a fold change ≥8 and *P* value < 0.05 to Gene Ontology (GO) and Kyoto Encyclopedia of Genes and Genomes (KEGG) enrichment analyses. GO term enrichment analysis revealed that ‘regulation of epithelial cell apoptotic process’, ‘peptide hormone processing’, ‘negative regulation of epithelial cell apoptotic process’ and ‘regulation of endothelial cell apoptotic process’ were the top four enriched terms (Figure S2B). Of note, KEGG pathway enrichment analysis revealed enrichment of genes involved in the complement and coagulation cascades (Figure S2C), and four genes associated with this pathway were upregulated in CAS knockdown cells (Figure 1I). Real‐time qPCR confirmed significant upregulation of complement component 3 (C3), complement factor I (CFI), fibrinogen gamma chain (FGG) and F2R‐like thrombin or trypsin receptor 3 (F2RL3) in CAS knockdown cells (Figure 1J). C3 is a well‐characterized component of the complement system. The complement system is a component of innate immunity that participates in the defence against invading pathogenic microorganisms and eliminates damaged host cells and cancer cells.^12^ Activation of the key players C3a and C5a attracts immune cells to sites of complement activation and triggers an inflammatory response.^13^ Interestingly, cancer cells also secrete complement proteins that regulate the tumour microenvironment and promote tumour growth and metastasis.^14^ In addition, TNBC cells have recently been shown to secrete complement proteins.^15^ Therefore, we further investigated the role of C3 in breast cancer with the Gene Expression Profiling Interactive Analysis 2 (GEPIA2) database and the Tumour Immune Estimation Resource (TIMER) database, a database for the analysis of tumour‐infiltrating immune cells. In contrast to CAS, which exhibited significantly increased expression in breast cancer, C3 was significantly downregulated in breast cancer (Figure 1K), and we identified a negative correlation of CAS expression with C3 expression in breast cancer in the TIMER database (Figure 1L). Lower expression of C3 was significantly associated with poorer overall survival and disease‐free survival (Figure 1M, Figure S2D). In addition, C3 expression was negatively correlated with tumour purity and positively correlated with infiltration of macrophages (*r* = 0.323, *P* = 2.96e‐25), neutrophils (*r* = 0.549, *P* = 6.83e‐76), dendritic cells (*r* = 0.566, *P* = 1.42e‐81), B cells (*r* = 0.389, *P* = 1.48e‐36), CD8+ T cells (*r* = 0.383, *P* = 2.08e‐35) and CD4+ T cells (*r* = 0.547, *P* = 3.66e‐76) (Figure S2E). These results suggest that the high C3 expression stemming from knockdown of CAS may attract immune cells to the tumour microenvironment.

In summary, we found that knockdown of CAS inhibited cell proliferation and metastasis as well as tumour growth, and we identified a novel inflammatory C3 pathway regulated by CAS in TNBC (Figure 1N). Interestingly, activation of C3 can promote or inhibit tumour progression in a cell type‐specific manner in cancers.^12^ The contrasting roles of C3 have also been observed in breast cancer: C3 deficiency in Her2/neu transgenic mice increased cancer metastasis, indicating that C3 exerts a tumour‐suppressive effect, while tumour‐derived C3 disrupted the blood–cerebrospinal fluid barrier and promoted cancer growth.^12^ In the present study, our results showed significant inhibition of cell proliferation after knockdown of CAS, accompanied by a significant increase in C3 expression in TNBC, indicating that C3 plays a tumour‐suppressive role in TNBC. In agreement with the experimental data, database analysis revealed downregulation of C3 in breast cancer, and lower C3 expression was associated with poor prognosis. Taken together, the present findings revealed the CAS‐dependent inflammatory C3 pathway in TNBC, which might provide new targets for TNBC therapy. Further studies are warranted to determine the detailed mechanism by which CAS regulates the C3 pathway in TNBC.

## CONFLICT OF INTEREST

The authors declare that there are no conflicts of interest.

## AUTHOR CONTRIBUTIONS

Hao Chen and Jianwu Shi conceived and designed the experiments. Mei Ye, Yufei Chen, Jianni Liu, Jiawei Tian and Xunda Wang performed the experiments and analysed the data. Mei Ye and Kin Lam Fok wrote the manuscript. Hao Chen, Kin Lam Fok and Jianwu Shi revised the manuscript.

## Supporting information


**Appendix** S1: Supplementary Materials and methods

**Figure S1 CAS is significantly upregulated in TNBC and required for TNBC cell growth.** (A) Comparison of CAS mRNA expression levels between TNBC and non‐TNBC samples in the GSE18864 dataset (Probe ID: 201112_s_at). (B) Comparison of CAS mRNA expression levels between TNBC and non‐TNBC samples in the GSE19615 dataset (Probe ID: 201111_at). An unpaired t test was used to analyse the data. (C) Cell viability assay to determine the effects of CAS knockdown in MDA‐MB‐231 cells. (D) Statistical analysis corresponding to Figure 1E. (E) Transwell assay was used to detect the migration and invasion of MDA‐MB‐231 cells after CAS knockdown. F‐G, Statistical analysis of the numbers of migrated (F) and invaded (G) cells 72 h after lentiviral transduction. (H) Growth curve of xenograft tumours

**Figure S2 RNA‐seq analysis of the downstream gene profile identifies C3 as a downstream target molecule after CAS knockdown.** (A) Volcano plot showing up‐ and downregulated genes with a fold change ≥2 and *P* value <0.05 after knockdown of CAS. (B) Gene Ontology enrichment analysis of differentially expressed genes with a fold change ≥8 and *P* value <0.05. (C) Kyoto Encyclopedia of Genes and Genomes pathway enrichment analysis of differentially expressed genes with a fold change ≥8 and *P* value <0.05. (D) Kaplan–Meier analysis of disease‐free survival for breast cancer patients with high (red line, *n* = 400) and low (blue line, *n* = 400) C3 mRNA expression as determined in the GEPIA2 database. (E) Correlation analysis of the C3 expression level and the infiltration levels of immune cells in the TIMER database

## Data Availability

The data that support the findings of this study are available from the corresponding author upon reasonable request.
