# Supplementary material for: Interfering with CSE1L/CAS inhibits tumour growth via C3 in triple‐negative breast cancer
Source: Cell Prolif. 2022 Apr 10;55(5):e13226. doi: 10.1111/cpr.13226 (PMC9136492; doi:10.1111/cpr.13226)
Supplement: Supplementary file 1 — Appendix S1: Supplementary Materials and methods [file CPR-55-e13226-s003.docx]

**Supplementary Materials and methods**

**Cell culture and lentivirus transduction**

MDA-MB-231 cells were cultured in DMEM supplemented with 10% FBS and incubated at 37℃ cell incubator containing 5% CO_2._ For lentivirus transduction, 2×10^4^ cells were plated into 6-well plates with 2 mL complete DMEM. After 12h culture, the lentivirus was used to transduction cells with 0.1% polybrene according to the manufacturer’s instructions. The lentivirus shRNA was purchased from Cyagen Biosciences and sequences are shNC CCT AAG GTT AAG TCG CCC TCG; shCAS GCA TGG AAT TAC ACA AGC AAA.

**Database analysis**

The GSE18864, GSE19615, GSE65194 datasets were downloaded from the GEO website(https://www.ncbi.nlm.nih.gov/geo/) and the CAS expression of breast cancer was compared between TNBC and non-TBNC. The C3 expression and survival analysis of breast cancer were also analyzed in GEPIA2. The correlations analysis between C3 expression and immune cell infiltration was performed in Tumor Immune Estimation Resource (TIMER) database (https://cistrome.shinyapps.io/timer/).

**Cell viability, EdU assay, and Colony formation assay**

For cell viability assay, cells were seeded in 96-well plates at a density of 2×10^3^ cells/well with 100uL complete medium supplemented plus 1.5ug/mL puromycin after transfection. The CCK-8 solution was added every 24 hours and the absorbance of OD450 nm was measured by a microplate reader after incubation for 2 hours. For the EdU assay, briefly, the EdU solution was added after removing the culture medium and incubated for two hours. Then fixed with 4% PFA and washed twice with PBS. Finally, the nucleus was labeled with Hoechst33342 and the pictures were photographed with a fluorescence microscope. For more details can refer to the manufacturer’s instruction of Cell-Light EdU Apollo 567 In Vitro Imaging Kit (Ribobio, Guangzhou, China). For colony formation assay, 500 cells were seeded into six-well plates and incubated for one week. Then the cells were fixed with 4% PFA and stained with crystal violet. After washing with ultrapure water twice, pictures were photographed with a camera, and colony numbers were counted using Adobe Photoshop software.

**Migration and invasion assay**

For cell migration assay: 2×10^4^ cells suspended in 500uL serum-free DMEM were seeded into the upper chamber of insert (Falcon® Cell Culture Inserts with 8.0 μm Transparent PET Membrane) and 600 μL of DMEM containing 10% FBS was added to the lower chamber. After incubation for 24 hours, the cells that migrated into the lower chamber were fixed with 4% PFA and stained with crystal violate. A cotton swab was used to wipe the upper chamber cells and inserts were photographed by an inverted microscope. For invasion assay: 2×10^4^ cells suspended in 500uL serum-free DMEM were seeded into the upper chamber of insert covered with matrigel (Corning® Bio-Coat™ Matrigel® Invasion Chambers with 8.0μm Transparent PET Membrane) and 600μL DMEM supplemented with 10% FBS was added into the lower chamber. After 24 hours, the cells that invaded the lower chamber were fixed and stained with crystal violate. After wiping the upper chamber cells, inserts were photographed by an inverted microscope. Migration and invasion cell numbers were counted using Adobe Photoshop software.

**Tumor xenografts**

Four weeks of female BALB/c nude mice were purchased from Jiangsu GermPharmatech Company and raised under specific pathogen-free conditions. After a week of acclimatization, 2×10^6^ MDA-MB-231 cells stably transfected with negative control (shNC) or shCAS lentivirus were injected into mice armpits. Tumor volume and weight were measured every two days and the tumor volume was calculated according to the formula: volume (mm^3^) = (length × width^2^)/2. After one month, the animals were sacrificed to harvest tumors.

**Western blot**

RIPA buffer supplemented with protease and phosphatase inhibitor was used to harvest cell and lysis on ice for 30 minutes. Then the cell lysates were centrifuged for 30 minutes at 12000 rpm to collect supernatant protein and protein concentration was measured by Pierce^TM^ BCA Protein Assay kit (23225, Thermo). The 30 ug total lysis was subjected to SDS-PAGE and transferred to PVDF membrane. Then the membrane was blocked with 5% nonfat dry milk or 5% BSA in TBST for one hour at room temperature and incubated with primary antibodies overnight at 4℃. After washing five times with TBST, the PVDF membrane was incubated with peroxidase-conjugated secondary antibody for one hour at room temperature. Subsequently, the membrane was detected by Pierce ECL Western Blotting Substrate (32209, Thermo). The primary antibodies’ information are as follows: Anti-CAS (ab70547, 1:1000, Abcam), Anti-CyclinD1 (2978, 1:1000, CST), Anti-CyclinB1 (12231T, 1:1000, CST), Anti-β-actin (A2228, 1:5000, Sigma), Anti-p53 (2524s, 1:1000, CST).

**RNA-seq and Quantitative Real-time PCR (qPCR) assay**

Total RNA from triplicates was extracted from 2×10^7^ MDA-MB-231 cells using TRIzol reagent (Sigma, USA). The bulk RNA-Sequencings (RNA-seq) was conducted in Majorbio Company (Majorbio, Shanghai, China) and data analysis was performed on the online platform of Majorbio Cloud Platform (www.major bio.com). For qPCR analysis, cDNA was synthesized with HiScript II Q RT SuperMix for qPCR (Vazyme, Nanjing, China) and qPCR was performed with SYBR dye ChamQ Universal SYBR Master Mix (Vazyme, Nanjing, China). The qPCR program was performed according to the manufacturer’s instructions. β-actin was used as an internal control. The 2^−ΔΔCt^ method was used to determine the relative gene expression level. The primers used were as follows:

| Primer | sequence |
| --- | --- |
| C3 | F:GGGGAGTCCCATGTACTCTATC; R: GGAAGTCGTGGACAGTAACAG |
| FGG | F:TTATTGTCCAACTACCTGTGGC; R:GACTTCAAAGTAGCAGCGTCTAT |
| CFI | F:CAGAGCCAGTAAAACTCATCGT; R:GGTTGGAATAGGTAAGGAGACCA |
| F2RL3 | F:GCTGCTGCATTACTCGGAC; R:ACGTAGGCACCATAGAGGTTG |
| β-actin | F:CATGTACGTTGCTATCCAGGC; R:CTCCTTAATGTCACGCACGAT |
